# Deep whole genome sequencing identifies recurrent genomic alterations in commonly used breast cancer cell lines and patient-derived xenograft models

**DOI:** 10.1186/s13058-022-01540-0

**Published:** 2022-09-24

**Authors:** Niantao Deng, Andre Minoche, Kate Harvey, Meng Li, Juliane Winkler, Andrei Goga, Alex Swarbrick

**Affiliations:** 1 Cancer Ecosystems Program, Garvan Institute of Medical Research, Sydney, Australia; 2 School of Clinical Medicine, Faculty of Medicine and Health, UNSW Sydney, Sydney, Australia; 3 Kinghorn Centre for Clinical Genomics, Garvan Institute of Medical Research, Sydney, Australia; 4 Department of Cell and Tissue Biology, University of California, San Francisco, San Francisco, CA USA; 5 Department of Medicine, University of California, San Francisco, San Francisco, CA USA

**Keywords:** Breast cancer cell lines, Patient-derived xenografts, Whole genome sequencing, Structural variants, Non-coding mutations

## Abstract

**Background:**

Breast cancer cell lines (BCCLs) and patient-derived xenografts (PDXs) are the most frequently used models in breast cancer research. Despite their widespread usage, genome sequencing of these models is incomplete, with previous studies only focusing on targeted gene panels, whole exome or shallow whole genome sequencing. Deep whole genome sequencing is the most sensitive and accurate method to detect single nucleotide variants and indels, gene copy number and structural events such as gene fusions.

**Results:**

Here we describe deep whole genome sequencing (WGS) of commonly used BCCL and PDX models using the Illumina X10 platform with an average ~ 60 × coverage. We identify novel genomic alterations, including point mutations and genomic rearrangements at base-pair resolution, compared to previously available sequencing data. Through integrative analysis with publicly available functional screening data, we annotate new genomic features likely to be of biological significance. *CSMD1*, previously identified as a tumor suppressor gene in various cancer types, including head and neck, lung and breast cancers, has been identified with deletion in 50% of our PDX models, suggesting an important role in aggressive breast cancers.

**Conclusions:**

Our WGS data provides a comprehensive genome sequencing resource of these models.

**Supplementary Information:**

The online version contains supplementary material available at 10.1186/s13058-022-01540-0.

## Background

Breast cancer cell line (BCCL) models are indispensable tools to study breast cancer biology and heterogeneity. Molecular profiling of BCCLs has generated useful insights into breast cancer subtypes and provides a resources for cancer gene discovery [2, 13, 19]. A number of large-scale cancer cell line projects have characterised hundreds of cell lines with whole transcriptome profiling [14], DNA microarray and targeted sequencing [3]. These studies provide great resources for cancer cell line studies; however, none of them have performed deep whole genome-wide sequencing analysis of genetic changes, including mutations and structural variations, on these critical breast cancer models.

Patient-derived xenograft (PDX) models, which closely resemble the heterogeneity of clinical BC, are established as important preclinical models [6, 8]. These breast cancer PDX models have been only characterised by shallow (< 1X coverage) whole genome sequencing (WGS) or exome sequencing [6].

WGS is the most sensitive method for detecting structural variants (SVs) and copy number variants (CNVs) and the only method to survey non-coding mutations. WGS is also a more powerful tool compared to exome-seq in detecting exome variants [4]. To fully understand the genomic features of these models, it is important to conduct deep WGS analysis of these models to exhaustively identify complex genomic features of PDX models. In this study, we performed deep WGS to provide a comprehensive resource of genomic events of these important BCCLs and PDX models.

## Results

### Whole genome sequencing of breast cancer cell line models

**Fig. 1:**
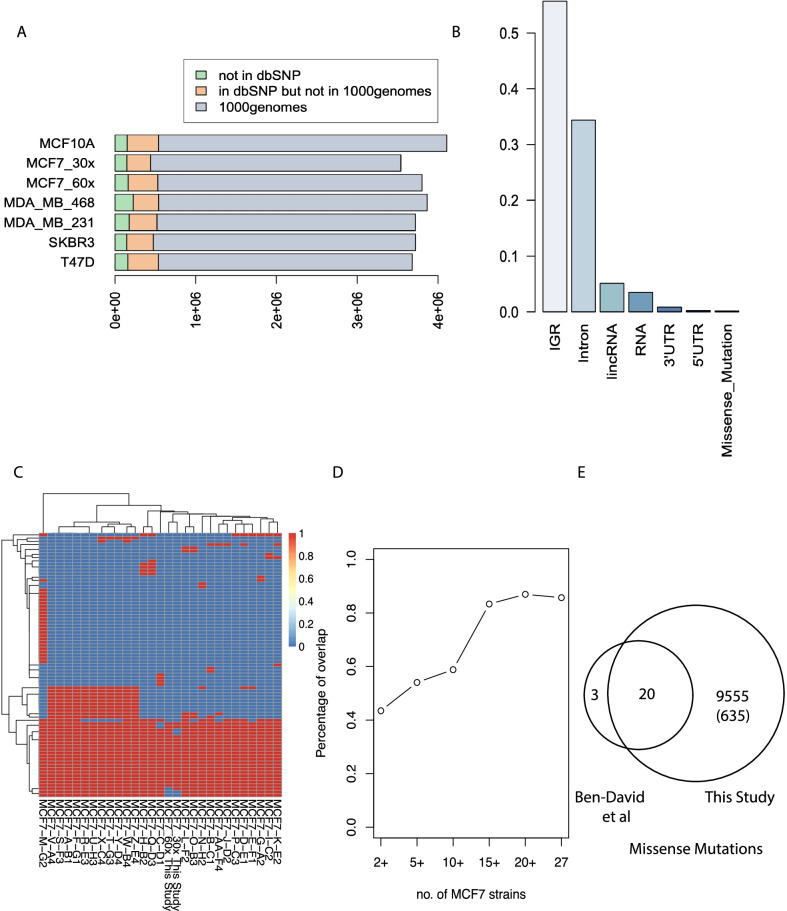
Summary of single nucleotide variations (SNVs) in six breast cancer cell lines, including two replicates for MCF-7. **A** Classification of SNVs into different categories: overlap with 1000 genome project; overlap with dbSNP but not in 1000 genome project; cell-line specific events; **B** Distribution of the SNVs in MCF-7 in respect to location of protein coding genes. “RNA variant” are the variants that lie on one of the RNA transcripts **C** unsupervised hierarchical clustering of MCF7 in this study together with 27 MCF7 strains in Ben-David et al., based on their missense mutation obtained from supplementary information from (Ben-David et al.) **D** Percentage of overlapping missense mutations between our findings and those identified in multiple strains of MCF7s in Ben-David et al. **E** Venn diagram shows the overlap of missense mutations in Ben-David et al. and this study

According to a PubMed search, the top six studied BCCLs are MCF7, MDAMB231, T47D, SKBR3, MCF10A and MDAMB468, which cover more than 90% of all BCCL associated studies across more than 90,000 publications (Additional file 1: Fig. S1). Previous studies on these cell lines are restricted to whole-exome or low-pass whole genome sequencing at 0.2X coverage [5]. Here we describe whole genome sequencing (WGS) of these models on the Illumina X10 platform with an average ~ 60 × coverage, including two replicates of the most-commonly used MCF7 cell line (~ 53× and ~ 78×). Raw reads were mapped to human genome GRCh37 and single nucleotide variants were called using the Issac pipeline [22]. WGS is the most accurate way to assess cell line identity [26]. We have compared the genotyping calls from our WGS data with SNP array from two independent studies previously published [3, 12]. Correlation analysis of the genotyping calls at the same genomic loci shows high concordance between ours and the published data (R > 0.9) (Additional file 1: Fig. S2), confirming the identity of all cell lines used in this study. In total, we have identified 3,540,312 to 4,108,844 variants per sample from the WGS data, including SNVs and small indels (Additional file 2: Table S1). The majority of variants are present in the dbSNP database [25] (94.0–96.2%) (Additional file 2: Table S1). About 90% of these dbSNP variants have also been reported in the 1000 genome projects [10], suggesting most variants in these cell lines models represents common variants in the human population, similar to the previous finding of WGS of the Hela cell line [15]. However, each of the cell lines has about 5% of total variants that are cell line specific variants (Fig. 1A).

In MCF7, for example, more than half of the cell line specific variants are located in inter-genic regions and ~ 1/3 are found in introns (Fig. 1B). This is similar across all the cell lines models (Additional file 1: Fig. S3). We compared the list of missense mutation in MCF7 to a recent study of multiple strains of MCF7s [5] (Fig. 1C). Most of the missense mutations in [5] shared between MCF7 strains, therefore likely to come from the founder tumor, were have been identified in this study (20/23), and the concordance with our data increases as the number of mutant MCF7 strains increases (Fig. 1D, E). In addition to the mutations reported in Ben-David et al., our WGS data identified 9,555 additional missense mutations, 635 of which are not reported in 1000 genome or dbSNP database (Additional file 2: Table S2). Sequencing of large cohorts of breast cancer tissue has revealed recurrent mutations in long non-coding genes including *MALAT1* and *NEAT1*[20]. Among all the cancer associated long non-coding genes reported [21] ([9], Lnc2Cancer v3.0), we identified mutations of *MALAT1*, *HOTAIR* and *ZFAS1* in these cell line models. For example, *Malat1* showed a heterozygous mutation (chr11:65271832T > C) in MCF7, but not in MDA-MB-231 (Additional file 1: Fig. S4). The complete list of variants in the non-coding regions in these cell lines models (Additional file 2: Table S3) could serve as a useful database for selection of models in non-coding RNA studies.

**Fig. 2:**
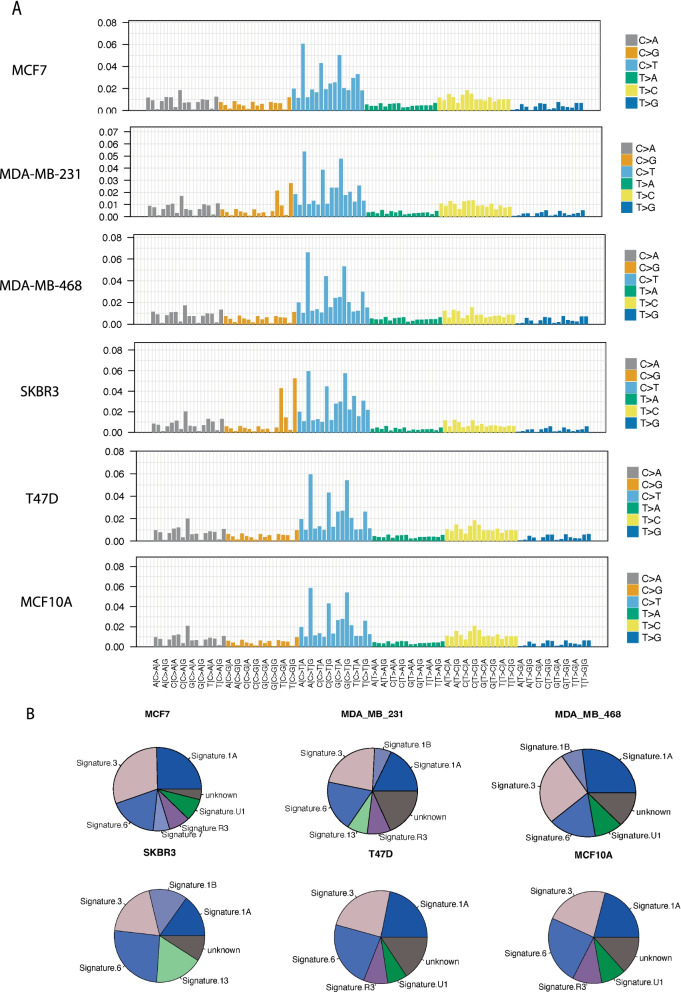
Mutational signature analysis of the Breast Cancer Cell Lines. **A** Output mutational profiles of the six cells lines from deconstructSigs displaying the fraction of mutations found in each trinucleotide context **B** pie charts of the mutational signatures identified for each of the six cell lines models from deconstructSigs

We also analysed the mutational signatures [2] using “deconstructSig” r package in the cell lines [24] (Methods). There is no difference between cell lines, likely due to the high number of mutations (Fig. 2A). As we do not have germline genomic data for cell line donors, we have used missense mutations for the cell lines after filtering against the dbSNP and 1000 genomes databases. Signatures 1A/1B, 3 and 6 are commonly observed across all the cells lines. Signature 1 has previously been commonly observed across all cancer types, while Signature 3 has been found in breast, ovarian and pancreatic cancers (Fig. 2B and Additional file 2: Table S4). Interestingly, Signature 3 is associated with DNA double-strand break-repair and germline and somatic BRCA1 and BRCA2 mutations. Signature 6 has been found in a majority of cancers and is associated with DNA mismatch repair and microsatellite unstable tumours.

**Fig. 3:**
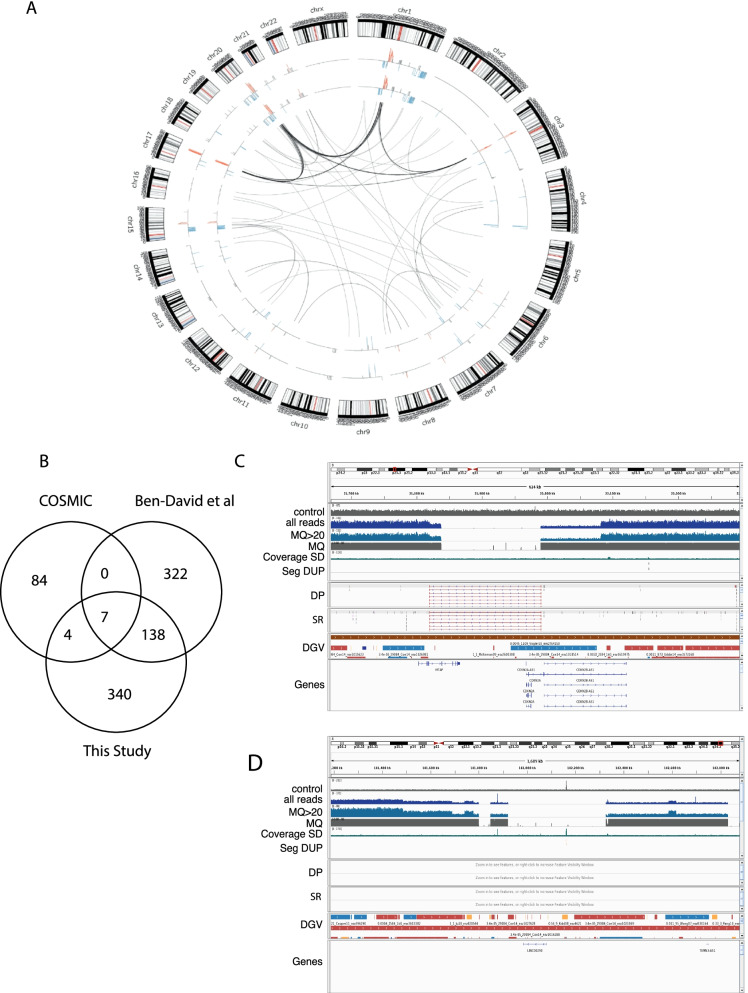
Genomics landscape of copy number and structural variants in breast cancer cell lines. **A** Representative circos plot of MCF7 for genomics alterations. Copy number events are summarized in the inner circle with red and blue colour indicates copy number gains and blue respectively, two inner circles are represented two replicates of MCF7 samples (one 30X and one 60X). Arcs connecting two loci of difference chromosomes indicate inter-chromosomal structural variations. **B** Venn diagram shows the overlap of CNV genes between COSMIC, Ben-David et al. and this study. **C** Representative genome browser view of copy number alterations covering a common CNV gene, CDKN2A. Tracks from top to bottom: depth of coverage in an NA12878 control (control), all reads in the sample (all reads), or reads with mapping quality >  = 20 (MQ > 20), the average mapping quality of aligned reads from the sample (MQ, if no reads align MQ = 0), coverage standard deviation from 500 controls (Coverage SD, indicating common CNV), overlapping segmental duplications published by Bailey JA et al. 2002 (SEG-DUP, used as control for germline CNVs), discordant pairs (DP), split reads (SR), variants from the Database of Genomic Variants (DGV), and RefSeq genes (Genes). **D** Representative genome browser view plot of a novel CNV gene LINC00290 in this study

In addition to the SNVs, we also called SVs and CNVs from the WGS data using different methods, including Breakdancer [7] and Delly [7] for structural variants; CNVnator [1] and Lumpy [1] for copy number (see Methods). A summary of SVs and CNVs identified in MCF7 and the other four cell lines is shown in Fig. 3A and Additional file 1: Fig. S5 respectively. In total, we have identified 321 inter-chromosomal SVs, with 38–108 SVs per cell line (Additional file 2: Table S5). We use the GREAT program [17] to perform pathway analysis of the SVs events from individual cell lines. As expected, the top enriched pathways are genes in amplified regions previously identified from breast cancer, such as genes like *ZNF217* and *BCAS3* in MCF7 [11]; a selected list of luminal genes have also been enriched in the luminal cell lines MCF7 and T47D (Additional file 1: Fig. S6).

In addition to SVs, we also identified copy number alterations in these cell line models (Additional file 3: Table S6). WGS has improved accuracy in detecting CNVs compared to exome-seq due to its uniform coverage [4]. We compared the CNVs identified from MCF7 with existing data from array-based COSMIC and shallow WGS from Ben-David et al. (Fig. 3B, Additional file 4: Table S7). Ben-David et al. reported that high variability in copy number calling, our study is more concordant with both of the studies than they are with each other (Fig. 3B). For example, a substantial proportion (138/478) of the CNVs identified in this study but not in COSMIC are reported in Ben-David et al. using shallow WGS, suggesting the array-based method missed a lot of CNVs due to poor coverage. WGS can identify the known key copy number events in each of the cell lines with fine resolution, including AURKA and MYC amplification, CDKN2A (Fig. 3C) deletion in MCF7, ERBB2 amplification in SKBR3, and CDKN2A deletion in MDAMD231 (Additional file 3: Table S6). Our WGS data can also identify novel CNVs not reported in either COSMIC or Ben-David et al. *LINC00290*, a long non-coding RNA, which has been previously reported to undergo copy number loss in a pan-cancer study (Zack et al. 2013), has a homozygous deletion in MCF7 (Fig. 3D). Furthermore, CNV boundaries were accurately detected by WGS compared to WES, in many cases with base pair precision (Additional file 3: Table S6). For example, in MCF7 cell line, the boundaries of the homozygous deletion cover only CDKNA2A, and the nearby CDKN2B is in a hemizygous deletion region (Fig. 3C). FOXA1 copy number gain is in a focal amplicon, whereas GATA3 is in a broad copy number gain region about 0.5 Mb long & ESR1 copy number gain is towards the 5’ end of the gene (Additional file 1: Fig. S7).

In order to estimate the functional impact of these variants, we compared our list of copy number variations with functional screen data from these cell lines [16]. In MCF7, 33 out of the 445 copy number events harbour at least one of the breast cancer essential genes from [16]. Interestingly this reveals coordinated amplification of *ESR1* and its co-factors *NCOA3* and *GATA3* and pioneer factor *FOXA1,* perhaps explaining the extreme estrogen-sensitivity of this cell line (Additional file 3: Table S6). Therefore, this study resolves known copy number events at nucleotide resolution and reports a substantial number of new copy number variants, some with evidence for function in these commonly used cell line models.

### Patient-derived xenograft models

**Fig. 4:**
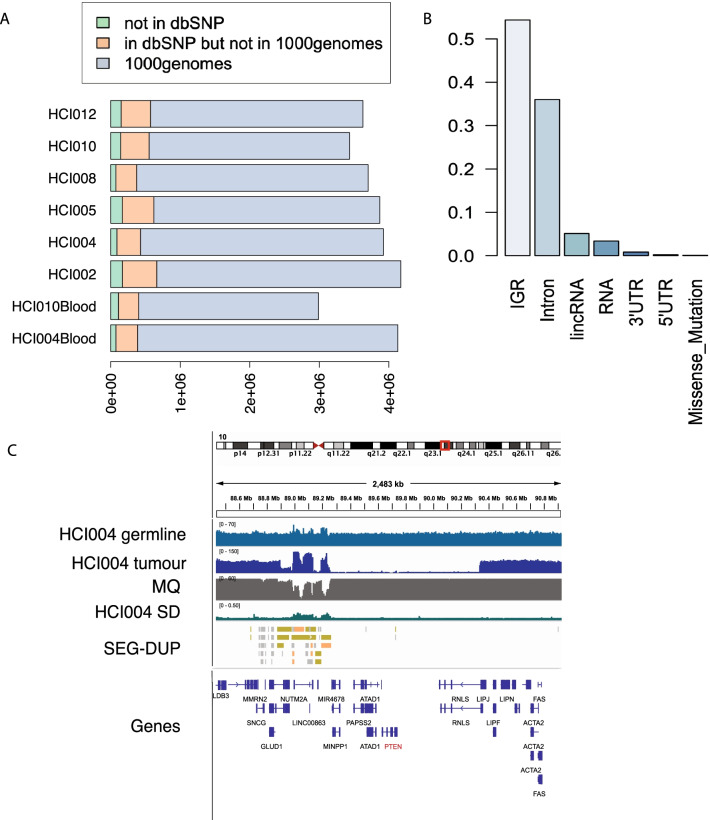
Summary of SNVs in breast cancer patient-derived xenograft models: **A** classifications of SNVs into three categories: overlap with 1000 genome project; overlap with dbSNP but not in 1000 genome project; PDX-specific variants. **B** Distribution of the SNVs in HCI002 model in respect to location of protein coding genes. **C** Representative genome browser view of copy number alteration covering *PTEN* in HCI004 model. Tracks from top to bottom: depth of coverage in HCI004 germline (control) and HCI004 PDX, and the average mapping quality of aligned reads from the sample (MQ, if no reads align MQ = 0), coverage standard deviation from 500 controls (Coverage SD, indicating common CNV), overlapping segmental duplications published by Bailey JA et al. 2002 (SEG-DUP, used as control for germline CNVs), discordant pairs (DP), split reads (SR), variants from the Database of Genomic Variants (DGV), and RefSeq genes (Genes)

**Fig. 5:**
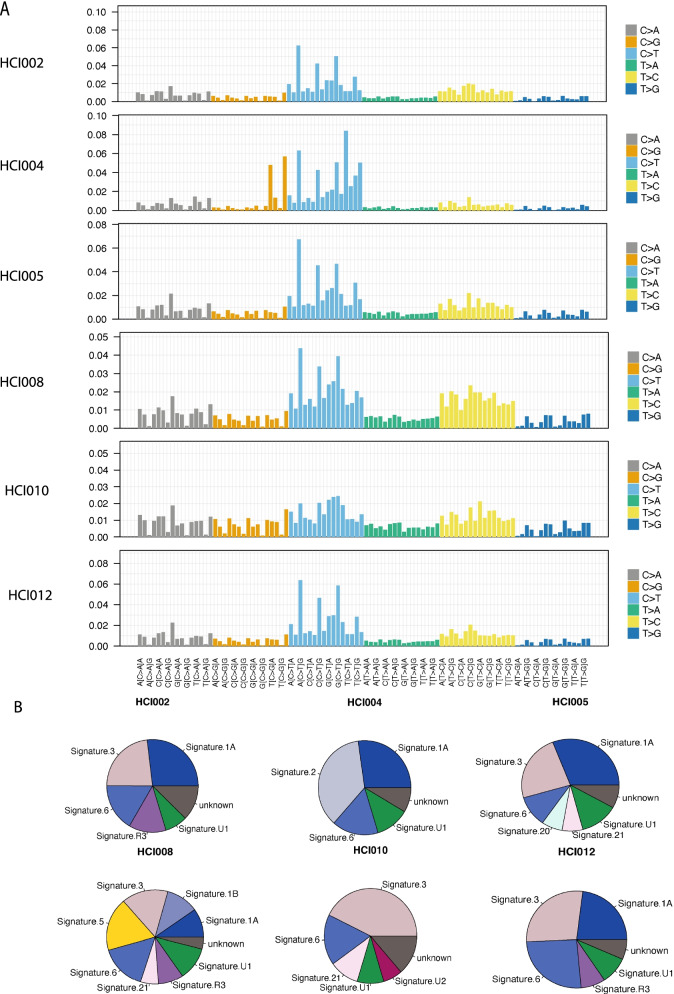
Mutational signature analysis of the PDXs. **A** Output mutational profiles of the six PDX models from deconstructSigs displaying the fraction of mutations found in each trinucleotide context **B** pie charts of the mutational signatures identified for each of the six PDX models from deconstructSigs

Bruna et al. performed shallow WGS (< 1X) profiling of 83 breast cancer PDX models and assessed their response to drug treatment [6]. Copy number calls were generated at 100 kb resolution. Here we have selected six well-established and frequently used PDX models with two matched blood samples [8] and performed WGS with at least 90 × coverage (blood samples were sequenced at 30 × coverage) (Additional file 5: Table S8) to generate reliable SNP, copy number and structural variant calls. Using the same pipeline as the cell line analysis based on the reads mappable to human genome, we identified 3,435,230 to 4,172,800 variants per sample from the WGS data, including SNVs and small indels (Additional file 2: Table S1). Similarly to the cell line data, the majority have been identified in the dbSNP database [25] (95.6–95.9%) (Additional file 1: Table S1). About 84% of these variants identified in dbSNP have also been reported in 1000 genome projects [10]. Each of the PDXs has about 4% of total variants as PDX specific variants (Fig. 4A). In HCI002, more than half of the PDX line specific variants are located in inter-genic regions, followed by variants in introns (Fig. 4B). While HCI004, HCI008 and two blood samples have the least number of variants in intron regions (Additional file 1: Fig. S8). To validate our findings, we performed exome sequencing analysis of selected models and identified There are 12 non-synonymous mutations identified using an independent exome sequencing analysis of these models, all of which have been also identified in our WGS data (Additional file 5: Table S9). Similar to the cell lines, we also performed mutational signature analysis in the PDX models. For PDX samples, we are able to generate somatic missense mutation lists for the HCI004 and HCI0010 models as we have matched blood samples for these models and can filter sample-specific germline variants in additional to 1000 genomes and dbSNP (Fig. 5A). While Signatures 1A/1B, 3 and 6 are found in most of the PDX models, there are also model-specific signatures (Fig. 5B). Interestingly, Signature 2, which is associated with hypermutation or kataegis, is found in the HCI004 model only. Other model-specific signatures including Signature 5 for HCI008 and Signature 3 for HCI010 and HCI012 (Additional file 5: Table S10).

Using a stringent cutoff [18], we have created a list of high confidence copy number alterations and structural variants for each of the PDXs (Additional file 6: Table S11 and Additional file 7: Table S12). Recurrently amplified regions across the PDX models are chromosome 8q in HCI002/005/010/012 (covering MYC, Additional file 8: Table S13) and chromosome 7p in HCI004/HCI008 (covering EGFR). Frequent deleted regions harbour classic tumour suppressor genes, for example PTEN in HCI004/HCI010 (Fig. 4C, Additional file 6: Table S11). There are a few SV regions that harbouring breast cancer associated tumour suppressor genes, such as PTPRD in HCI008 model and CSMD3 in HCI012 model (Additional file 7: Table S12).

**Fig. 6:**
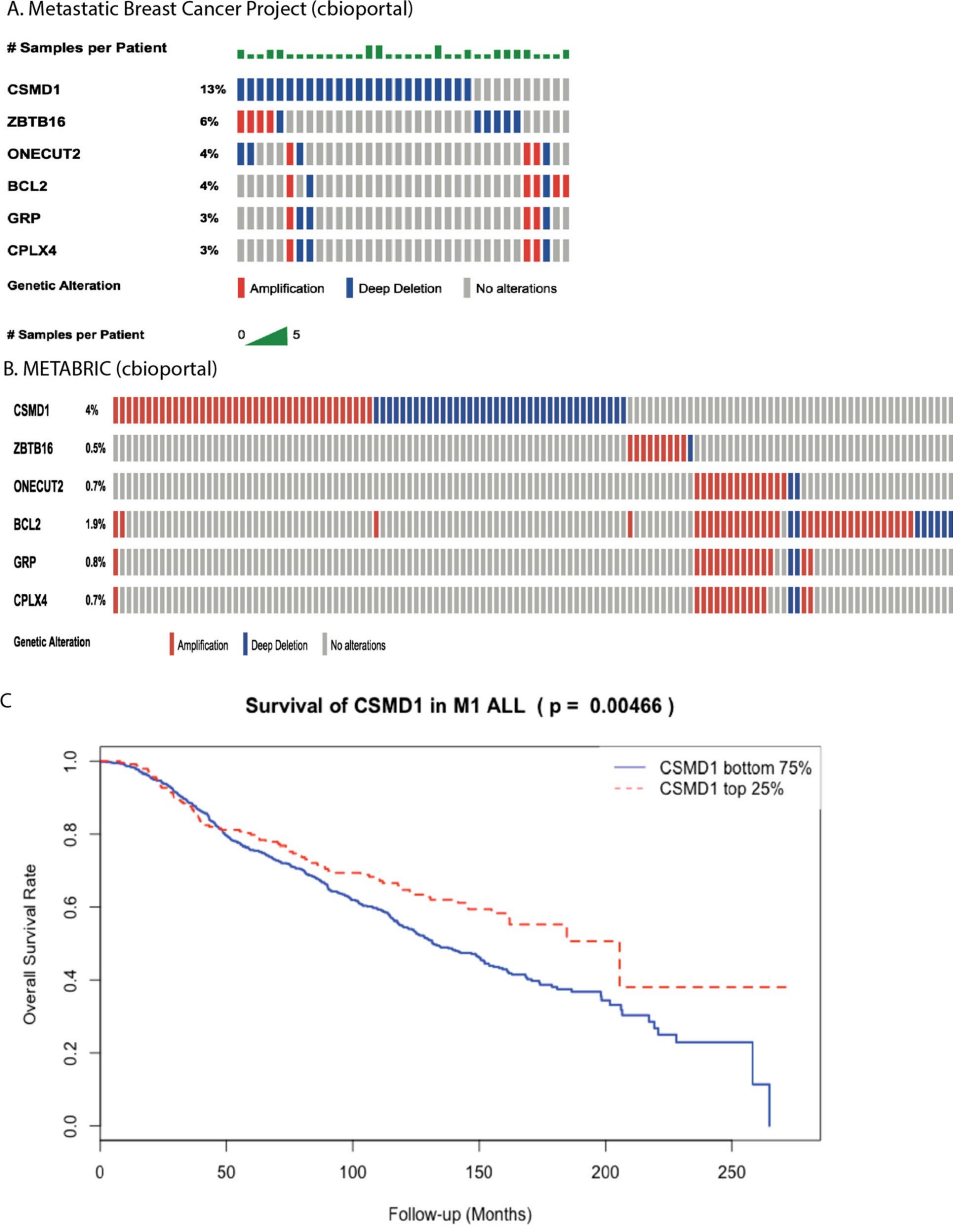
Genes with enriched genomic alterations in PDXs. **A**, **B** Genes with frequent copy number alterations in PDXs samples showing higher copy number variations in a metastatic breast cancer cohort than a primary breast cancer cohort (cbioportal). Each row indicates a gene and each column indicates a breast cancer sample. Copy number gains are in red and deletions are in blue. CSMD1, for example, showing a much high frequency of copy number deletion in the metastatic cohort. **C** Kaplan–Meier survival analysis of METABRIC discovery cohort samples stratified by CSMD1 expression status. Top 25% samples with high CSMD1 is in red, showing a better survival outcome compared to those with low CSMD1 expression

Because PDX tumours probably model more aggressive breast cancers (DeRose et al.), we proposed that genomic analysis of PDX models may reveal genes associated with very aggressive disease. Interestingly, the most frequently deleted gene in PDX models, *CSMD1*(in 3 out of our six PDX models), is much more frequently deleted in metastatic breast cancer (14%) than in early disease (2%) (Fig. 6A). Low expression of *CSMD1* is also associated with poor survival outcome in the METABRIC early cancer cohort (Fig. 6B) and is especially associated with poor survival outcome in the LumB subtype (Additional file 1: Fig. S9). Interestingly, we identified copy number loss of CSMD1 in 23 of 32 PDXs in another PDX sequencing dataset [6]. These data suggest that *CSMD1* plays a critical role in suppressing growth or survival of metastatic breast cancers.

## Discussion

We have completed whole genome analysis for widely used breast models, including mutations, copy number and SVs. The data has refined previously finding in cell lines such as *AURKA* and *MYC* amplification/*CDKN2A* deletion in MCF7, ERBB2 amplification in SKBR3, and *CDKN2A* deletion in MDAMD231 at nucleotide resolution. Our WGS data also identified novel mutations in non-coding genes and novel CNVs such as a homozygous deletion in *LINC00290*, and will be an important resource for research in this area. It is worth noting that WGS didn’t identify all the mutations reported in the targeted sequencing from Ben-David et al. (Fig. 1D). This could be due to that targeted sequencing provides very high coverage (> 300x) for these regions and can identify mutations with very low minor allele frequency. Since WGS is far more accurate in identifying CNVs boundaries, it can provide further insights into well-known cancer associated genes, such as focal amplification of *FOXA1* in MCF7 versus broad copy number gain in *GATA3* and partial gain of *ESR1*. By comprehensively identifying the genomic features of these models, the field can now choose appropriate models to examine the functional significance of genes or pathways of interest.

Genomic studies of PDX may help us identify genomic features associated with very poor prognosis, as growth in a PDX acts as a ‘filter’ to enrich for the most aggressive tumours and cells that otherwise occur at low patient frequency in unselected breast cancer cell populations. In support of this, we found recurrent deletion of *CSMD1* in the PDXs models and its associated with metastatic disease and survival. However, since we are comparing unmatched cohorts of primary and metastatic disease and relying on different platform to detect copy number, further studies on the match primary and metastatic disease based on the same platform is needed to confirm the roles of CSMD1 in metastatic breast cancer. Further extension of this idea to more PDX models may reveal further drivers of aggressive disease. The WGS data in this study is a valuable resource for other genomics studies to map to, for instance, CHIP-Seq or RNA-Seq studies to focus on non-coding and regulatory regions.

## Conclusions

We have applied deep whole genome sequencing (WGS) of commonly used cell lines and PDX models using the Illumina X10 platform with an average ~ 60 × coverage. We show that this resource can be used to identify novel genomic alterations, including point mutations and genomic rearrangements, compared to previously available sequencing data.

Specific outcomes include:

- A comprehensive list of point mutations, copy number and structural variants for cell lines and PDX models.
- Integrative analysis with publicly available functional screening data identifies new genomic features of biological significance.
- CSMD1, a known tumour suppressor gene, identified with deletion in 50% of our PDX models, suggesting an important role in aggressive breast cancers.
- Raw data and processed data are publicly available through European Genome Archive.

## Methods

### Sample acquisition and preparation

The MCF7 cell line was originally obtained from Michigan Cancer Foundation, the MCF10A from Brugge Lab, Boston, the MDAMB231 from EG&G Mason RI Worcester MA, and the MDAMB468, SKBR3 and T47D were from ATCC. Cells were cultured in their standard media to expand and frozen cell pellets used for DNA extraction. Breast Cancer PDX models were obtained from Alana Welm, and were expanded in house using techniques previously reported in DeRose et al. (2010).

### DNA extraction

The DNeasy Blood & Tissue Kit (Qiagen) was used for DNA isolation from about 25 mg frozen tumor, according to the manufacturer’s recommendations. RNase A (Qiagen) was used to obtain RNA-free genomic DNA. Only isolated DNA with A260/280 and A260/230 ratios above 1.8 and proven to be high quality by gel electrophoresis were used for sequencing.

### Whole genome sequencing analysis of cell lines and PDX

The five cancer cell lines MCF7, MDAMB231, T47D, SKBR3, MDAMB468 and one non-malignant cell line MCF10A and six PDX models were submitted for sequencing with at least 60 × coverage. Raw reads were mapped to human genome GRCh37 using Issac aligner from Illumina and called point mutations and small indels by Issac (Issac aligner and variant caller v1.14 were used). Oncotator was used to annotation the variants, details of the definitions for each variant category can be found from oncotator web page (https://gatk.broadinstitute.org/hc/en-us/articles/360041848811-Funcotator). Copy number alterations from the cell lines were estimated from cn.mops (R package), and CNVnator [1]. Structural variants were computed from Breakdancer [7], Delly [7] and Lumpy [1]. Breakdancer and Delly were used to call large inter-chromosomal changes, while lumpy and CNVnator were performed to call small scale SVs and CNVs. The SVs and CNVs from different tools were grouped together for each individual model. Structural variants were annotated and visualized using ClinSV (https://github.com/KCCG/ClinSV) [18] and IGV [23]. Raw sequencing files (fastqs) and annotated variants files are deposited at European Genome Archive with accession number EGAS00001006285.

### Mutational signature analysis

*deconstructSig r package version 1.8.0* was used to analyse the mutation signatures. “*whichSignatures*” function was used to identify the mutational signatures with signature score default cutoff at 0.06 and the signature plots were plotted using “*plotSignature*” and “*makePie*” functions.

### Cell line identity check using SNP array

These common cell lines have been profiled by Affymetrix SNP 6.0 array previously in multiple studies. Raw cel files were downloaded from the Heiser et al. and CCLE and were analysed by affymetrix genotyping console software. Correlation analysis between the genotyping calls between array and WGS data to confirm cell line identity. The coefficient correlation was calculated based on concordance rate of genotyping calls between difference platform. For example, *V*_*cij*_, *V*_*dij*_ are the number of concordant and discordant variants between platform i and platform j, then the coefficient correlation is define as $Cij=\frac{Vcij}{Vcij+Vdij}$.

## Supplementary Information

**Additional file 1: Fig. S1**. number of citations from PubMed for breast cancer cell lines. Number of citations for each of the cell lines obtained from PubMed, data retrieved from Pubmed in May 2022. **Figure S2.** Heatmap of correlation of genotyping calls of breast cancer cell lines from three different studies. Correlation analysis of genotyping calls from this study to compare with SNP calls from Heiser et al and CCLE. **Figure S3**. barplots of number of different types of variants identified in the cell lines. **Figure S4**. IGV plot of a non-coding gene MALAT1 in MCF7 and MDAMB231 respectively, showing a mutation in MCF7 but not in MDAMB231. **Figure S5**. Circos plot of structural variations in breast cancer cell lines, MDA-MB-231, T47D, MDA-MB-468 and SKBR3. Arcs connecting two loci of difference chromosomes indicate inter-chromosomal structural variations. **Figure S6**. GREAT analysis of genes affected by SV variants in the breast cancer cell lines. **Figure S7**. Representative IGV plot showing copy number gains in FOXA1, GATA3 and ESR1 in MCF7. Tracks from top to bottom: depth of coverage in an NA12878 control (control), all reads in the sample (all reads), or reads with mapping quality >=20 (MQ>20), the average mapping quality of aligned reads from the sample (MQ, if no reads align MQ=0), coverage standard deviation from 500 controls (Coverage SD, indicating common CNV), overlapping segmental duplications published by Bailey JA et al. 2002 (SEG-DUP, used as control for germline CNVs), discordant pairs (DP), split reads (SR), variants from the Database of Genomic Variants (DGV), and RefSeq genes (Genes). **Figure S8**. barplots of number of different types of variants identified in the six PDX models. **Figure S9**. Kaplan-Meier survival analysis of METABRIC samples stratified by CSMD1 expression status by four different breast cancer PAM50 subtypes. Top 25% samples with high CSMD1 is in red, showing a better survival outcome compared to those with low CSMD1 expression. (PDF 8335 kb).

**Additional file 2: Table S1**. Summary of total number of variants in difference cell line models and PDXs from whole genome sequencing. **Table S2**. List of the 635 additional missense mutations in MCF7 compared to Ben-David et al. **Table S3**. The complete list of variants in the non-coding regions in these cell lines models. **Table S4**. Weights of mutational signatures identified from DeconstructSigs for each of the cell lines. **Table S5**. List of structural variants identified in the cell line models.

**Additional file 3: Table S6**. List of copy number alterations identified in the cell line models.

**Additional file 4: Table S7**. Comparison of the genes with copy number alterations in COSMIC, Ben-David et al and this study.

**Additional file 5: Table S8**. Summary of sequencing depth for PDXs. Table S9: List of mutations identified and validated in breast cancer PDXs. Table S10: Weights of mutational signatures identified from DeconstructSigs for each of the PDX models.

**Additional file 6: Table S11**. List of copy number alterations and identified in the PDXs.

**Additional file 7: Table S12**. List of structural variants identified in the PDXs.

**Additional file 8: Table S13**. List of genes with common copy number alterations in the PDXs.

## Data Availability

All data analysed in this study are included in this article and its supplementary information files. The raw sequencing data supporting these findings are available at European Genome Archive (accession number EGAS00001006285).
