# Supplementary material for: Deep whole genome sequencing identifies recurrent genomic alterations in commonly used breast cancer cell lines and patient-derived xenograft models
Source: Breast Cancer Res. 2022 Sep 24;24:63. doi: 10.1186/s13058-022-01540-0 (PMC9509640; doi:10.1186/s13058-022-01540-0)
Supplement: Supplementary file 1 — Additional file 1: Fig. S1. number of citations from PubMed for breast cancer cell lines. Number of citations for each of the cell lines obtained from PubMed, data retrieved from Pubmed in May 2022. Figure S2. Heatmap of correlation of genotyping calls of breast cancer cell lines from three different studies. Correlation analysis of genotyping calls from this study to compare with SNP calls from Heiser et al and CCLE. Figure S3. barplots of number of different types of variants identified in the cell lines. Figure S4. IGV plot of a non-coding gene MALAT1 in MCF7 and MDAMB231 respectively, showing a mutation in MCF7 but not in MDAMB231. Figure S5. Circos plot of structural variations in breast cancer cell lines, MDA-MB-231, T47D, MDA-MB-468 and SKBR3. Arcs connecting two loci of difference chromosomes indicate inter-chromosomal structural variations. Figure S6. GREAT analysis of genes affected by SV variants in the breast cancer cell lines. Figure S7. Representative IGV plot showing copy number gains in FOXA1, GATA3 and ESR1 in MCF7. Tracks from top to bottom: depth of coverage in an NA12878 control (control), all reads in the sample (all reads), or reads with mapping quality >=20 (MQ>20), the average mapping quality of aligned reads from the sample (MQ, if no reads align MQ=0), coverage standard deviation from 500 controls (Coverage SD, indicating common CNV), overlapping segmental duplications published by Bailey JA et al. 2002 (SEG-DUP, used as control for germline CNVs), discordant pairs (DP), split reads (SR), variants from the Database of Genomic Variants (DGV), and RefSeq genes (Genes). Figure S8. barplots of number of different types of variants identified in the six PDX models. Figure S9. Kaplan-Meier survival analysis of METABRIC samples stratified by CSMD1 expression status by four different breast cancer PAM50 subtypes. Top 25% samples with high CSMD1 is in red, showing a better survival outcome compared to those with low CSMD1 expression. (PDF 8335 kb). [file 13058_2022_1540_MOESM1_ESM.pdf]

Figure S1

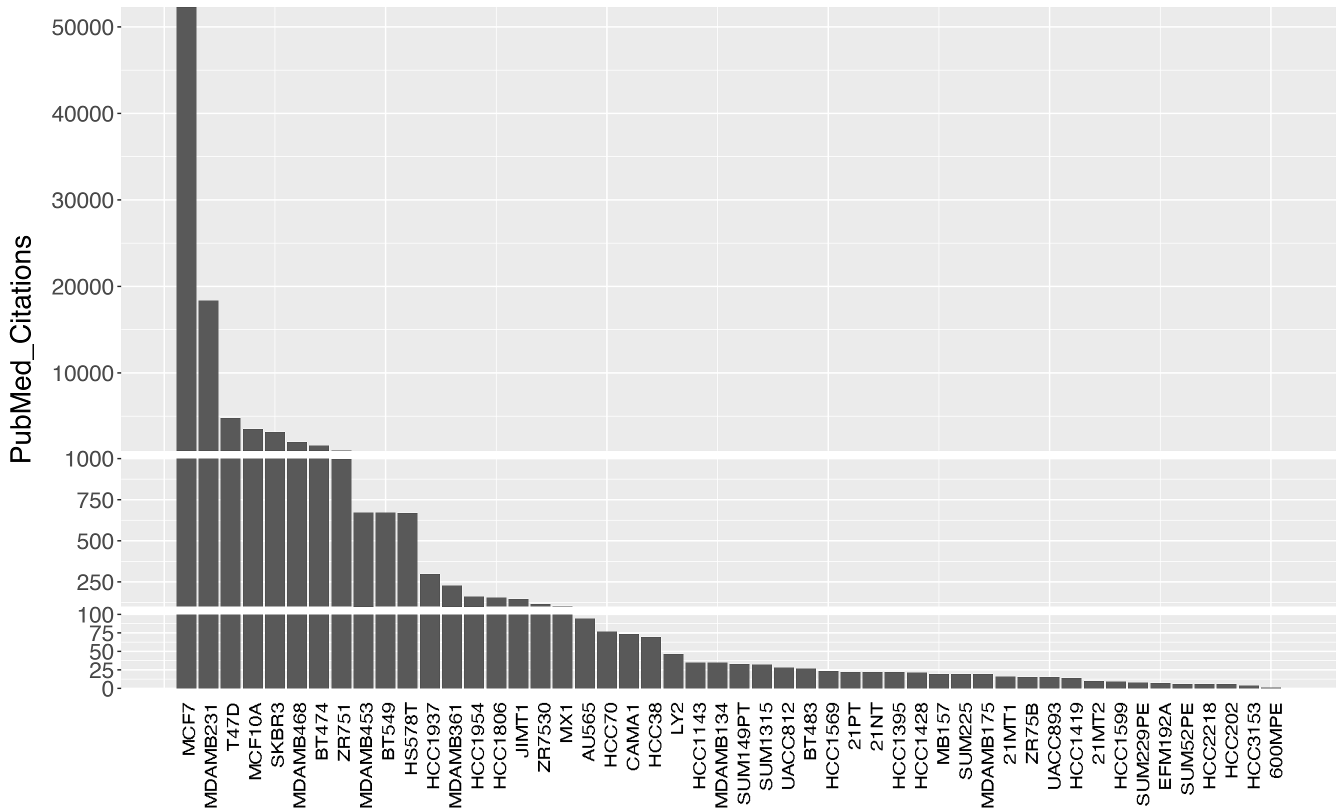

Figure S2

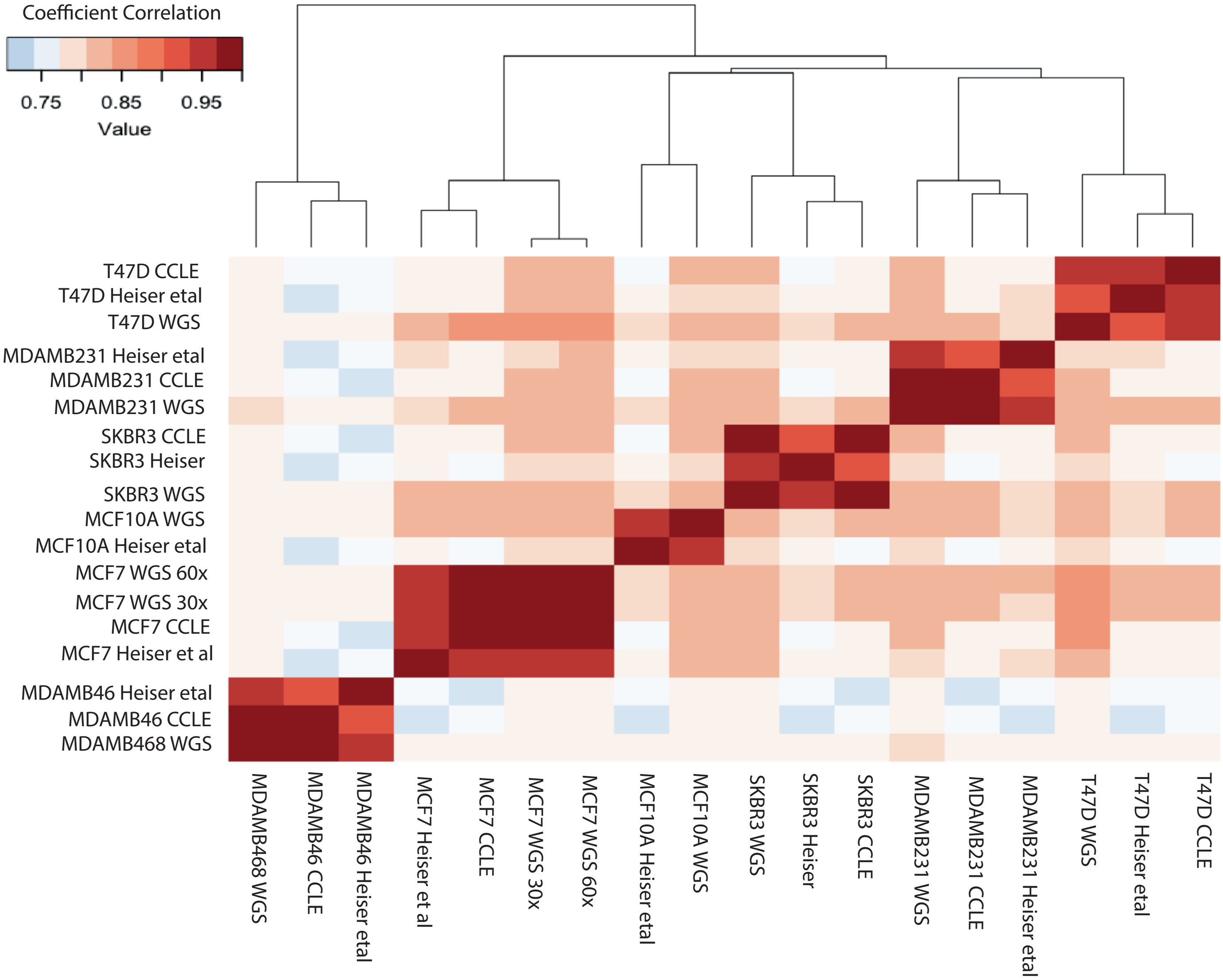

Figure S3

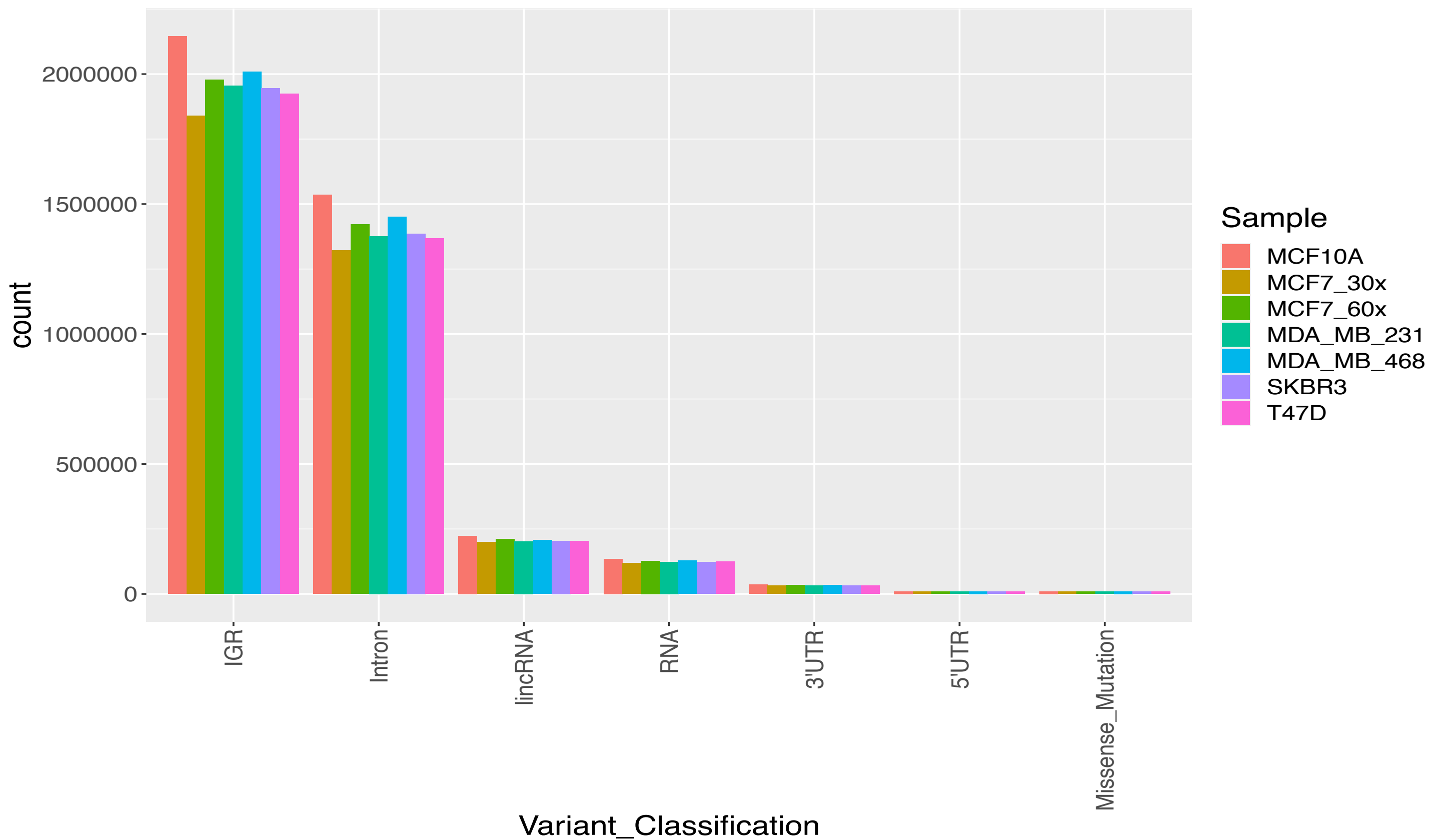
count
2000000
1500000
1000000
500000
0
IGR
Intron
lincRNA
RNA
3'UTR
5'UTR
Missense_Mutation
Variant_Classification
Sample
MCF10A
MCF7_30x
MCF7_60x
MDA_MB_231
MDA_MB_468
SKBR3
T47D

Figure S4

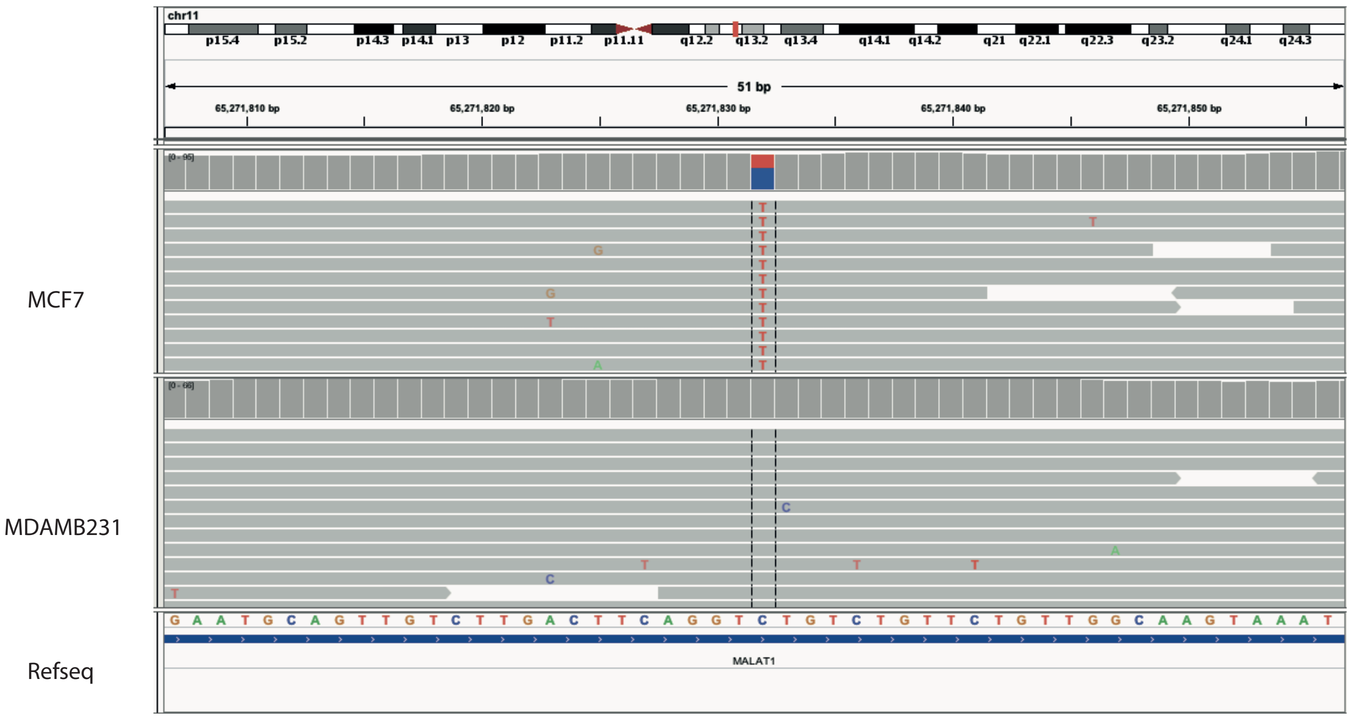

Figure S5

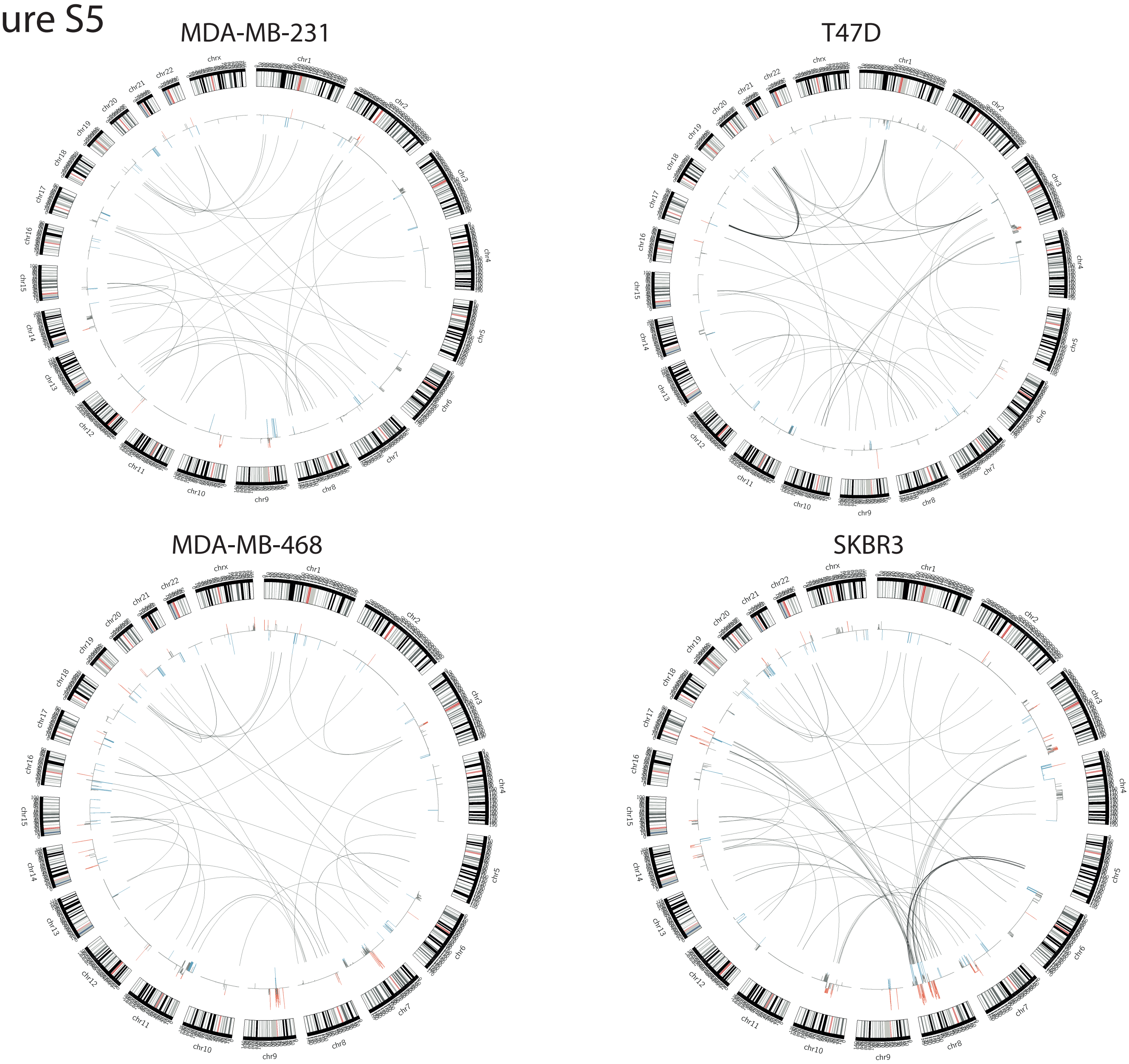

Figure S6

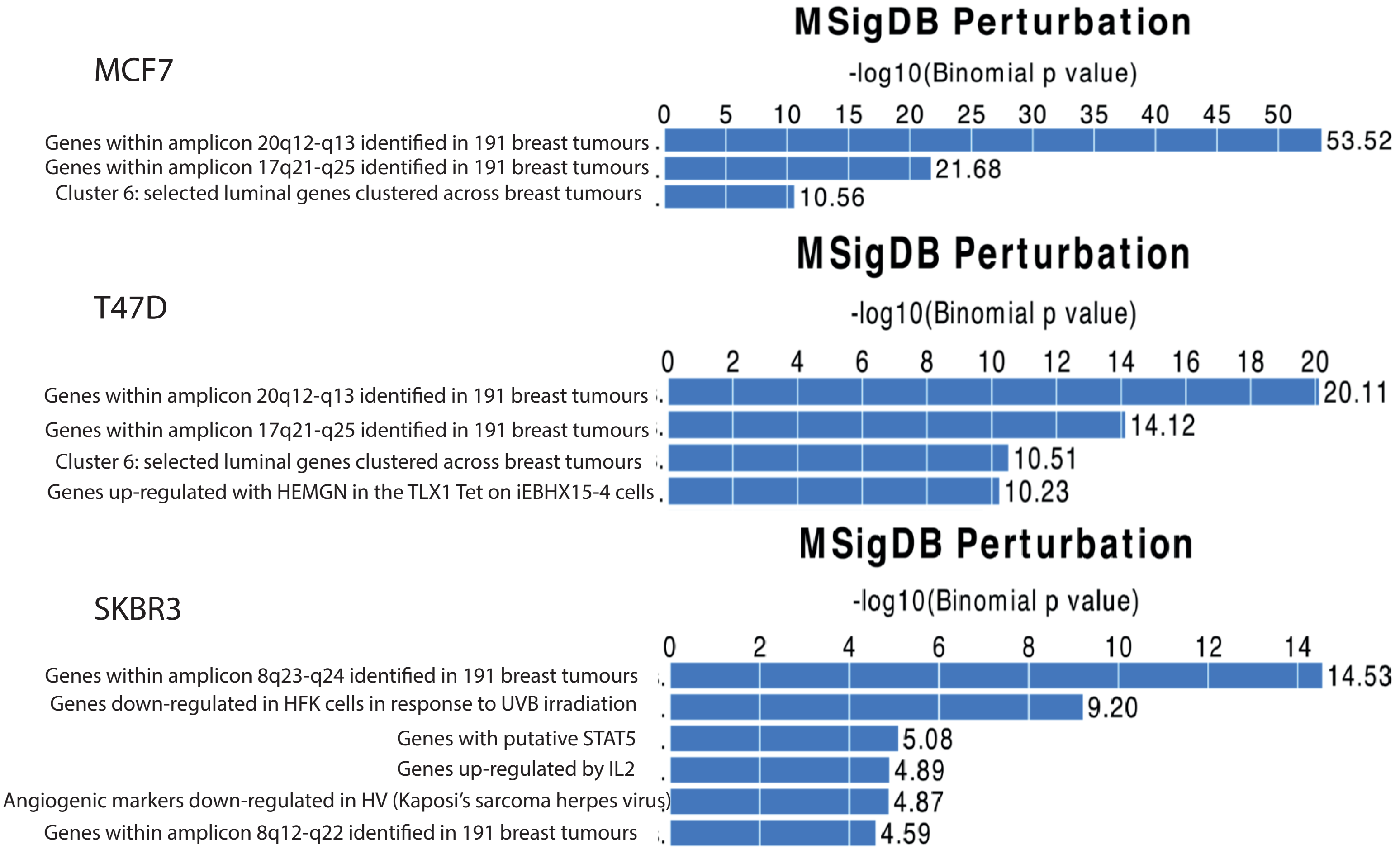
MCF7
MSigDB Perturbation
-log10(Binomial p value)
0 5 10 15 20 25 30 35 40 45 50
Genes within amplicon 20q12-q13 identified in 191 breast tumours
53.52
Genes within amplicon 17q21-q25 identified in 191 breast tumours
21.68
Cluster 6: selected luminal genes clustered across breast tumours
10.56
T47D
MSigDB Perturbation
-log10(Binomial p value)
0 2 4 6 8 10 12 14 16 18 20
Genes within amplicon 20q12-q13 identified in 191 breast tumours
20.11
Genes within amplicon 17q21-q25 identified in 191 breast tumours
14.12
Cluster 6: selected luminal genes clustered across breast tumours
10.51
Genes up-regulated with HEMGN in the TLX1 Tet on iEBHX15-4 cells
10.23
SKBR3
MSigDB Perturbation
-log10(Binomial p value)
0 2 4 6 8 10 12 14
Genes within amplicon 8q23-q24 identified in 191 breast tumours
14.53
Genes down-regulated in HFK cells in response to UVB irradiation
9.20
Genes with putative STAT5
5.08
Genes up-regulated by IL2
4.89
Angiogenic markers down-regulated in HV (Kaposi's sarcoma herpes virus)
4.87
Genes within amplicon 8q12-q22 identified in 191 breast tumours
4.59

Figure S7

FOXA1- 8 copies, focal amplification

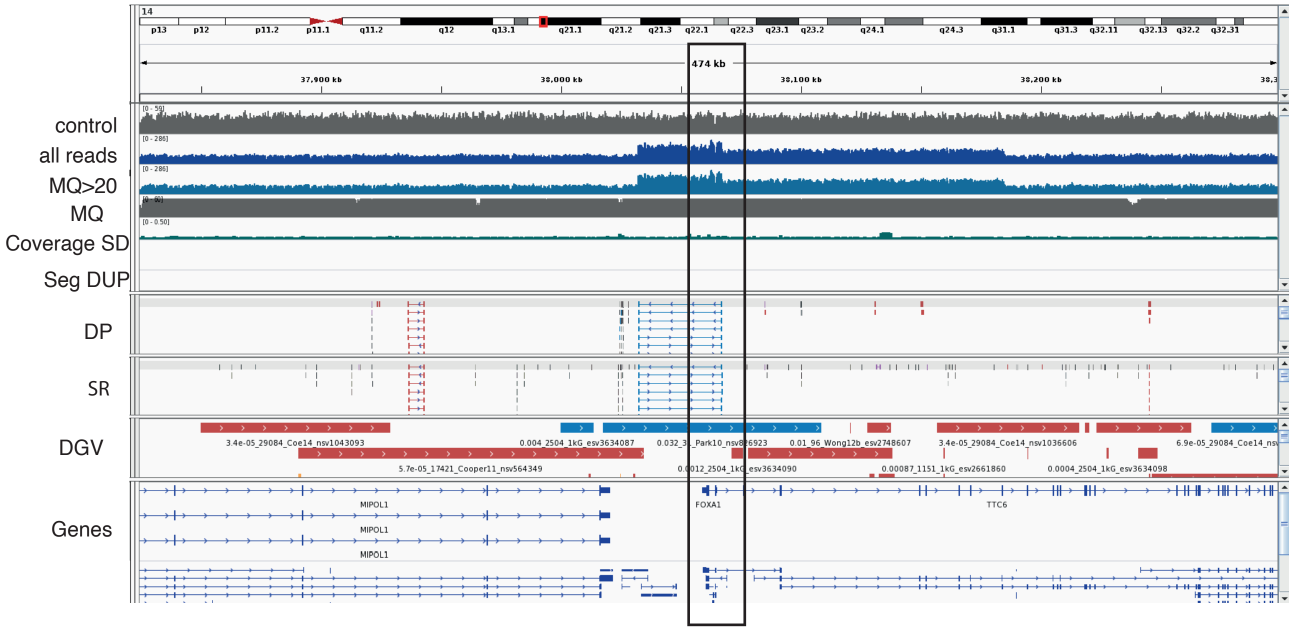

GATA3, broad gain, 4 copies

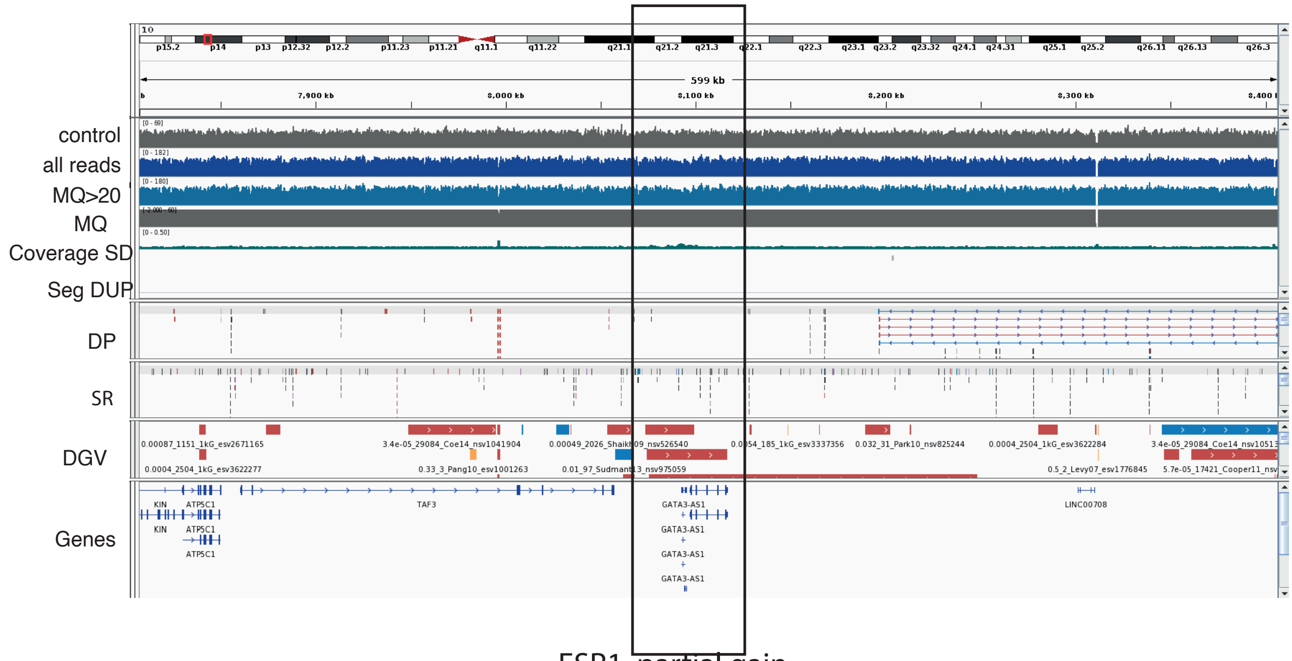

ESR1, partial gain

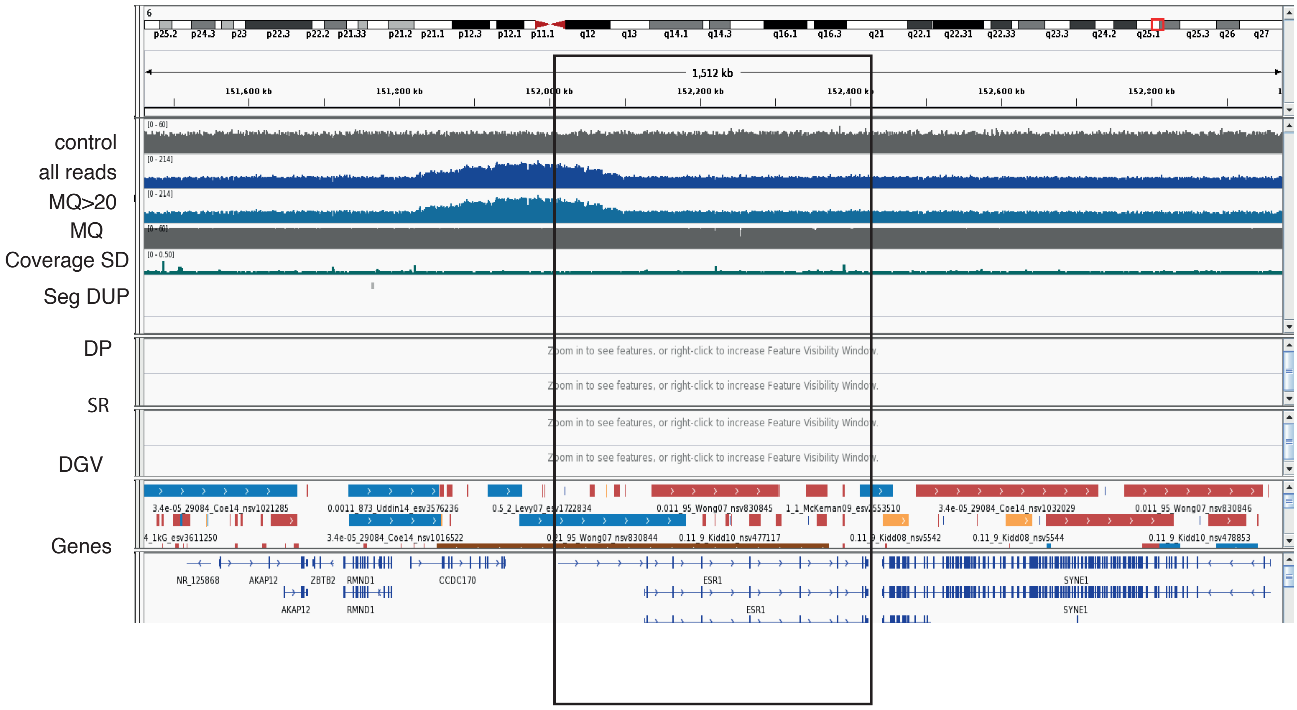

Figure S8

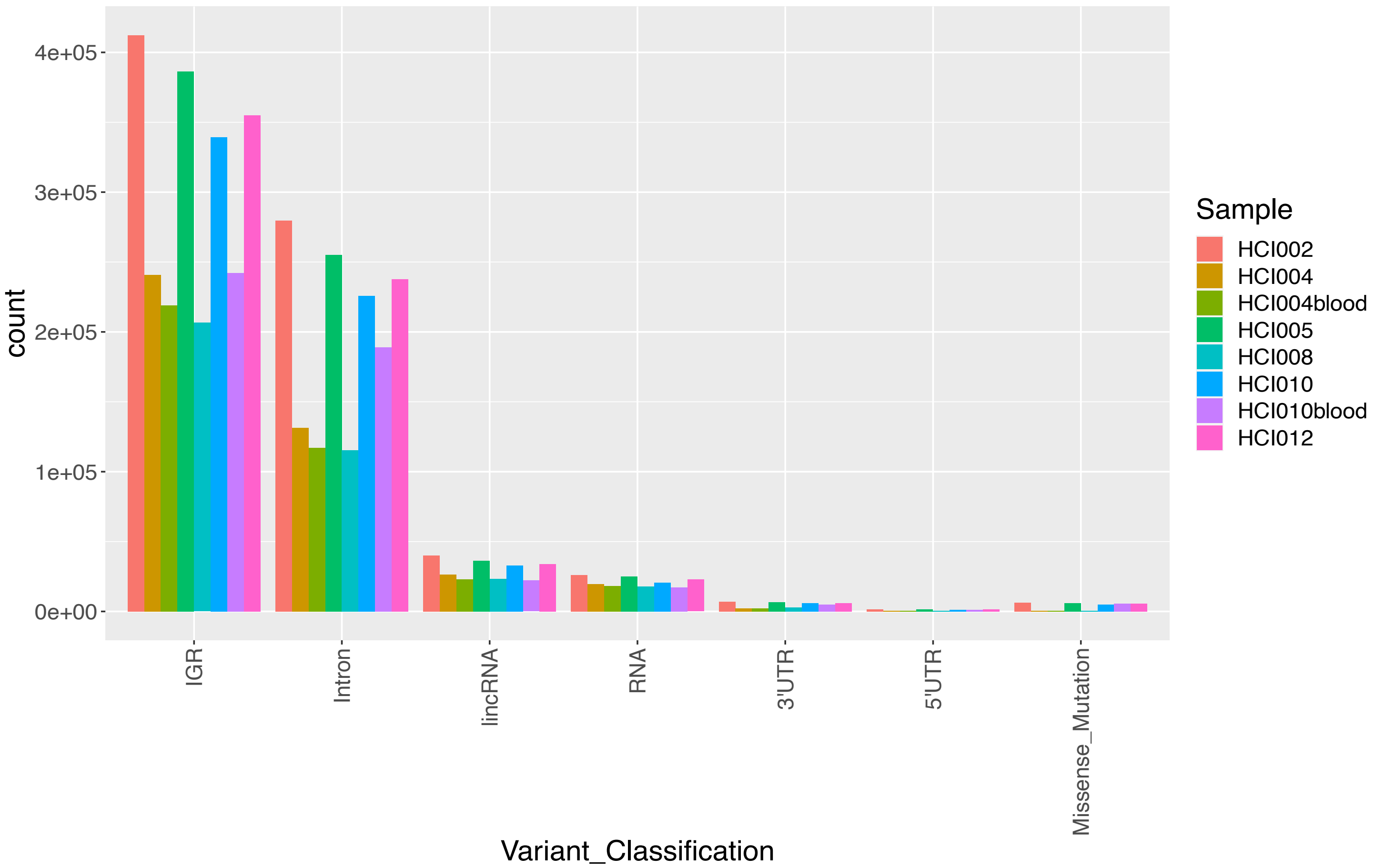

4e+05
3e+05
2e+05
1e+05
0e+00
count
IGR
Intron
lincRNA
RNA
3'UTR
5'UTR
Missense_Mutation
Variant_Classification
Sample
HCI002
HCI004
HCI004blood
HCI005
HCI008
HCI010
HCI010blood
HCI012

Figure S9

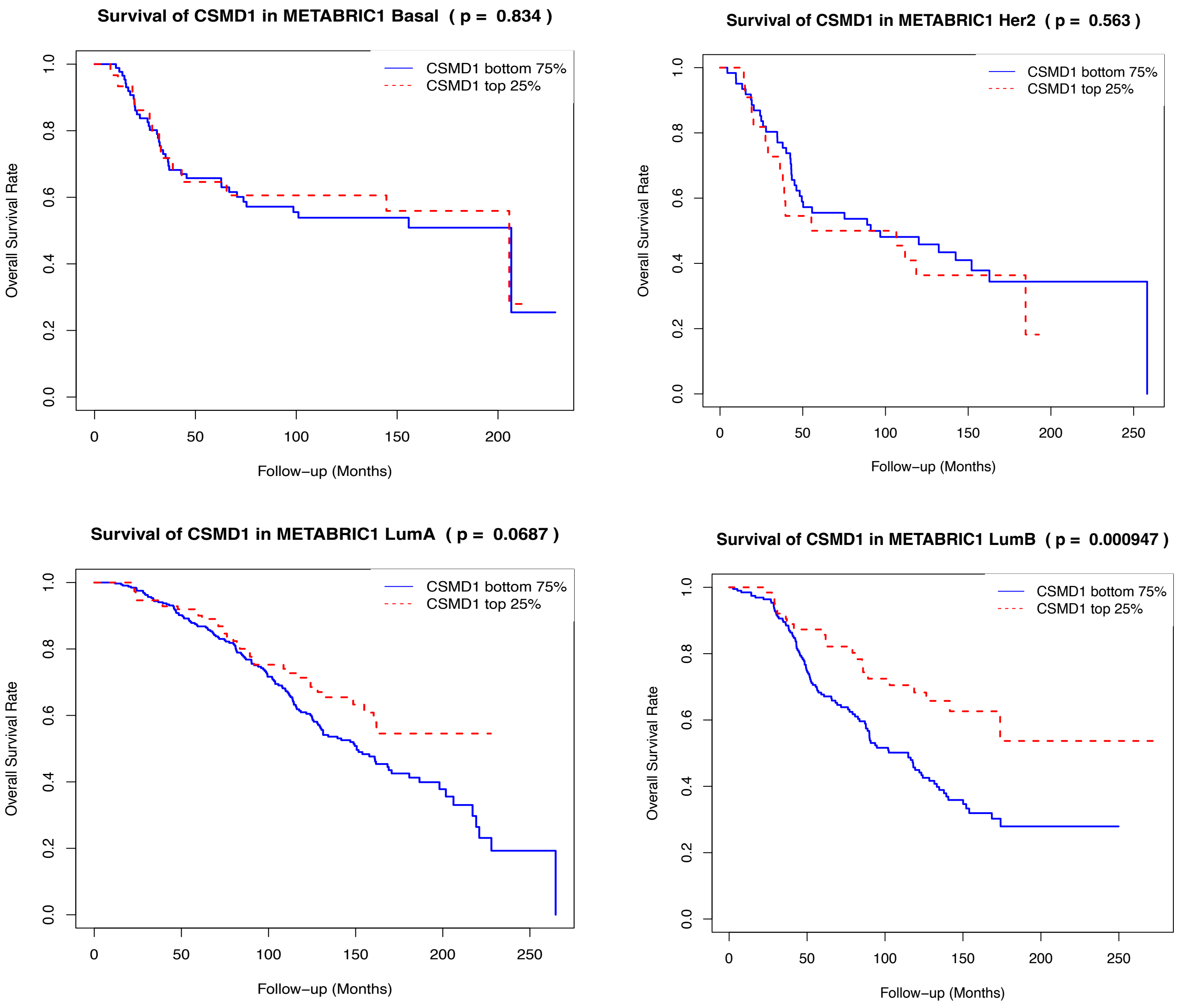
Survival of CSMD1 in METABRIC1 Basal (p = 0.834)
CSMD1 bottom 75%
CSMD1 top 25%
Overall Survival Rate
Follow−up (Months)
Survival of CSMD1 in METABRIC1 Her2 (p = 0.563)
CSMD1 bottom 75%
CSMD1 top 25%
Overall Survival Rate
Follow−up (Months)
Survival of CSMD1 in METABRIC1 LumA (p = 0.0687)
CSMD1 bottom 75%
CSMD1 top 25%
Overall Survival Rate
Follow−up (Months)
Survival of CSMD1 in METABRIC1 LumB (p = 0.000947)
CSMD1 bottom 75%
CSMD1 top 25%
Overall Survival Rate
Follow−up (Months)
